# Recruiting older people at nutritional risk for clinical trials: what have we learned?

**DOI:** 10.1186/s13104-015-1113-0

**Published:** 2015-04-15

**Authors:** Cynthia Piantadosi, Ian M Chapman, Vasi Naganathan, Peter Hunter, Ian D Cameron, Renuka Visvanathan

**Affiliations:** Discipline of Medicine, University of Adelaide, Adelaide, SA 5000 Australia; Centre for Education and Research on Ageing, University of Sydney, Concord Hospital, Concord, NSW 2139 Australia; Alfred Health, Caulfield Hospital, 260, Caulfield, VIC 3162 Australia; Adelaide Geriatrics Training and Research with Aged Care (GTRAC) Centre, School of Medicine, University of Adelaide, Paradise, SA 5075 Australia; John Walsh Centre for Rehabilitation Research, Sydney Medical School, University of Sydney, Sydney, NSW 2065 Australia; Aged and Extended Care Services, The Queen Elizabeth Hospital, Woodville South, SA 5011 Australia

**Keywords:** Malnourished, Frail elderly, Patient recruitment, Randomized control trial, Frail

## Abstract

**Background:**

The difficulty of recruiting older people to clinical trials is well described, but there is limited information about effective ways to screen and recruit older people into trials, and the reasons for their reluctance to enrol. This paper examines recruitment efforts for a community-based health intervention study that targeted older adults.

**Methods:**

One year randomized control trial. Undernourished men and women, aged ≥ 65 years and living independently in the community were recruited in three Australian states. Participants were allocated to either oral testosterone undecanoate and high calorie oral nutritional supplement or placebo medication and low calorie oral nutritional supplementation. Hospital admissions, functional status, nutritional health, muscle strength, and other variables were assessed.

**Results:**

4023 potential participants were identified and 767 were screened by a variety of methods: hospital note screening, referrals from geriatric health services, advertising and media segments/appearances. 53 participants (7% of total screened) were recruited. The majority of potentially eligible participants declined participation in the trial after reading the information sheet. Media was the more successful method of recruiting, whereas contacting people identified by screening a large number of hospital records was not successful in recruiting any participants.

**Conclusion:**

Recruitment of frail and older participants is difficult and multiple strategies are required to facilitate participation.

**Trial registration:**

Australian Clinical Trial Registry: ACTRN 12610000356066 date registered 4/5/2010

## Background

The global population is aging, with the annual growth rate of persons aged 80 years or over (3.8%) currently twice that of persons over 60 years of age (1.9%) [1]. A major challenge is to provide good health care for older people. To achieve this, relevant clinical evidence is required. However, older people, in particular the frail, are difficult to enrol in clinical research studies [2].

Patient recruitment is usually one of the most challenging aspects of clinical research studies [3], and there are particular difficulties involved in enrolling older adults [2,4], who are more likely than young adults to have factors which might exclude them from studies, such as coexistent chronic diseases, cognitive impairment, limited mobility and polypharmacy [5]. Recruitment and retention of frail, older people is resource intensive [6,7]. Higher costs associated with transportation and time commitment can also be specific barriers to the recruitment of frail and older people [3,8,9]. Because of these challenges, many intervention studies of older people target individuals who have fewer co-morbidities, are less frail, are on fewer medications, and with less disability than their peers who will be subsequently treated with the study treatment if found beneficial.

Some researchers have described reasons for non-participation in research studies and how these might be overcome [10,11]. The use of flexible recruitment protocols using multiple methods probably enhances recruitment in primary care studies [12].

In order to ensure that recruitment to future studies of older people is improved, it is important for researchers to share their experiences of challenges and success. Therefore, in this paper we describe the challenges, process and outcome of recruiting older people at-nutritional risk to a multi-centre, community based, randomized control trial looking at the efficacy of interventions that include a nutritional supplement and pharmaceutical agent [13].

## Methods

The protocol for this randomized control trial of daily oral testosterone and nutritional supplement drink versus placebo in community dwelling older people at-risk of under-nutrition is described in detail elsewhere [13]. Detail relevant to recruitment is provided in this paper. The hypothesis, based on positive results in a pilot study [14], was that this combined anabolic treatment would reduce the rate of hospital admissions, an important clinical endpoint, in this vulnerable group of older people. As part of the study information provided, and as a possible incentive to take part, potential participants were made aware that the combined treatment had been associated with a significant reduction in the rate of hospital admissions in the pilot study.

### Participants

#### Inclusion and exclusion criteria

In this study, the participants were said to be at-risk of under-nutrition if they fulfilled the following:

- a Mini Nutritional Assessment (MNA) score between 17 and 23.5 [15]; and
- a body mass index (in kg/m^2^) of <22 or a self-reported weight loss of ≥7.5% in the 3 months before enrolling in the study.

Given that this trial included a pharmaceutical agent, the following exclusion criteria were applied:

- the inability to comply with the protocol;
- Folstein’s Mini Mental State Examination score ≤23 [16];
- Elevated hematocrit (>50%); history of prostate cancer, prostate-specific antigen (PSA) concentrations greater than the age-related normal range, or an irregular prostate on examination;
- a history of breast cancer in men and women;
- preexisting androgenic signs or symptoms of concern (deep voice, hirsutism, acne, or androgenic hair loss) in women;
- significant depressive symptoms using the Geriatric Depression Scale (short form) score ≥11 [17];
- cardiac failure corresponding to New York Heart Association class III and above;
- Myocardial infarction or stroke within the past 12 months, unstable angina, coronary artery procedure (stent, angioplasty or coronary artery bypass grafting) within the past 12 months, unstable arrhythmia (does not include controlled atrial fibrillation);
- uncontrolled hypertension; systolic blood pressure >170 mmHg and/or diastolic blood pressure >100 mmHg
- abnormal liver function tests (alanine aminotransferase, *γ*-glutamyltransferase, bilirubin, or alkaline phosphatase >2 times the upper limit of normal);
- Estimated creatinine clearance < 30 ml/min (by the equation of Baracskay and Jarjoura for ambulatory elderly participants [Cr clearance = 4.4/serum creatinine (mmol/L) + (88-age)] AND/OR serum creatinine concentration > 0.2 mmol/l. [18]
- any disease that, in the opinion of the investigator, is likely to lead to death within 1 year;
- testosterone or other androgen therapy within 4 months of starting the study; and
- women on oestrogen or hormone replacement therapy that have not been on a stable dose for the last 3 months.

### Recruitment methods

Recruitment occurred in the three Australian states of South Australia, Victoria and New South Wales using several different methods:

- Direct referrals from the geriatric and other health-care services. Investigator clinicians and other geriatrician/gerontology, allied health colleagues could refer consenting participants from their rehabilitative, ambulatory or outreach services (inpatient or ambulatory) for further contact and review by the research officer. Potential participants recently in hospital could be enrolled into the study 3 months after the last hospitalization and once health status was stabilized.
- Media and Advertisements. Television (major free-to-air channel evening news medical reports), website, radio (2 appearances by investigators on Australian Broadcasting Commission programs), newspaper advertisements (papers with state-wide and local coverage, including in special sections and supplements directed at older citizens) and presentations at public meetings related to the health of older people and attended by potential participants. Advertisement shown in Appendix 1.
- Flyers; and
- Case note review by the research officer. Medical records (Concord and Caulfield Hospital) were screened for the following criteria: age ≥65 years, English as primary language and no history of breast and prostate cancer. Where an individual expressed interest in the study from advertisements (media or flyers), following referral from a health care service clinician, they were contacted by telephone and underwent an initial screening (phone screening). This included some preliminary screening questions (i.e. recent weight loss and estimated weight and height). They were also provided some brief information about the study. If participants appeared eligible, information about the research study was posted by the research officer to interested participants. For those identified by case note review information sheets were posted to participants before further contact. Participants were then contacted by telephone the week after by a research officer to determine willingness to take part and for initial phone screening as described above.

Reasons for participant ineligibility or unwillingness to take part determined at the phone screening were documented. For participants still eligible and interested in participating after this phone screen, the research officer organized a visit at home or other location, at the participant’s convenience, to complete the study procedures including written consent and blood collection.

Recruitment first commenced in South Australia in March 2010 as it had previously conducted a similar pilot study [14]. Victoria followed, recruiting from August 2011 and New South Wales from February 2012. Subject recruitment ended in June 2013 due to expiry of available time and resources.

### Recruitment target

The sample size was based on power calculations performed on the results of the pilot study [14]. To have a power of 90% to detect a significant (at P = 0.05 [2-sided]) difference in the number of days of hospitalisation (the primary end-point) between the treatment groups 28 participants per group were required, and to have a power of 90% to detect a significant (at P = 0.05 [2-sided]) difference in the number of participants with non-elective admissions 30 participants per group were required. Given the desire to also investigate the secondary end-point of cost-effectiveness through quality of life years, a greater target of 200 participants was set (100 per group).

### Ethics statement

The intervention study was approved by the Human and Research Ethics Committee of the Queen Elizabeth Hospital (Adelaide, South Australia), Concord Hospital (Sydney, New South Wales) and Caulfield Hospital (Melbourne, Victoria). The intervention study was also registered with the Australian Clinical Trial Registry: ACTRN 12610000356066 and has been previously described in detail [13]. The study protocol has previously been reported [13].

## Results

**Figure 1:**
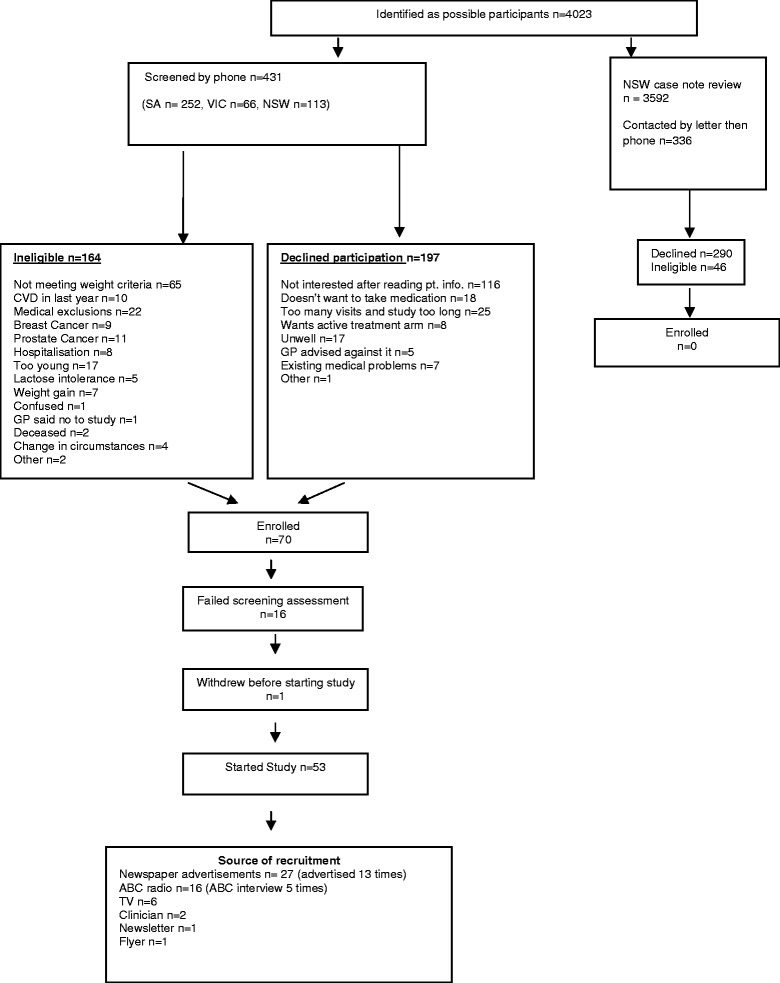
Flow diagram of patient recruitment.

An overview of the recruitment process is shown in Figure 1.

### Enrolment rate

Four thousand and twenty three potential participants were identified. The majority of these (3592 [89%]) were identified by case note review in New South Wales, and of these 336 met initial inclusion criteria regarding age and co-existent medical conditions, and were sent written material about the study. These subjects and an additional 431, largely identified in response to newspaper advertisements, radio and TV appearances by the investigators, and in a few cases after referral by friends or others, were then contacted for screening by phone (total n = 767).

At screening, 210 (27%) of the 767 participants were found to be ineligible, while 487 (64%), declined to take part. Detailed reasons for potential New South Wales participants’ identified by case note review ineligibility or declining to take part are not available. Reasons for ineligibility and decision to decline participation for the other 431 potential participants are detailed in Figure 1. The most common reasons for ineligibility were not meeting the weight criteria (40%) and co-existent exclusionary medical conditions (32%), including cardiovascular disease, prostate cancer and breast cancer. The majority of those who declined to take part (58%) did so for non-specific reasons after reading the information sheet. Potential participants often commented or seemed to suggest to the research officer at screening that they did not think they were malnourished (even when apparently satisfying the study weight criteria for this), while others seemed to be put off by the length and detail of the information sheet. A variety of other specific reasons were given for declining to participate, including being unwell at the time of screening, not wishing to take additional medications and advice from general practitioners and others.

Fifty four participants were recruited and consented. One (recruited after referral by a friend) withdrew just before commencing the study and 53 started the study. Twenty seven (51%) were recruited through newspaper advertisements, with radio program appearances by investigators (n = 16, 30%) and TV segments also contributing (n = 6, 11%). Two subjects were recruited as a result of referral by health care professionals. No subjects were recruited as a result of case note reviews.

## Discussion

In spite of many factors militating against inclusion of older adults to this randomized, placebo-controlled trial, we were able to recruit some older participants at-risk of under-nutrition, many of whom were frail. However, despite intensive recruiting and screening using a variety of methods, for four years, in the capital cities of three Australian states, we were not able to recruit the desired number of 200 undernourished/at risk older participants.

Recruitment methods that were most effective in this study were newspaper advertisements and appearances by investigators on talk-back radio programs. Websites, mailings, fliers, and referrals by health-care professionals were generally ineffective for recruitment of older adults. Screening hospital records and then contacting possible eligible participants so-identified was without success in recruiting participants. A higher recruitment rate may have been achieved by trying some other strategies such as directly approaching older people through senior community groups which can be time consuming [19], or by providing incentives to general practitioners to identify eligible participants from their practice. For example, Ellish et al. [20], investigated enrolment yields by type of activity and type of venue in older African Americans into a behavioural intervention study designed to increase eye examination. Highest enrolment rates were word of mouth (69%), flyers (67%) and senior centres (66%). In another study, Forster et al. [21] invited older people to a six-month randomised controlled dietary intervention which aimed to explore the relationship between diet and immune function. In their study, the most successful recruitment method was contacting recruits by letter on GP headed note paper using contacts provided from General Practice (90%).

It seems likely that difficulties we had recruiting participants were due to both the details of our particular study, and to difficulties in recruiting older, particularly unwell and frail participants for any research study. Factors related to this particular study probably contributed substantially to the low recruitment rate. Many potential participants were ineligible on the basis of co-existent medical conditions and not meeting the low body weight inclusion criteria for under-nutrition or risk of under-nutrition. The restrictions resulting from stringent inclusion and exclusion criteria for randomised controlled trails particularly limit recruitment in studies of older people. Masoudi et al. [22], have reported similarly low recruitment rates for clinical trials of heart failure in older people. The oldest patients and women are particularly unlikely to meet trial criteria.

More than half of potential participants we contacted declined to take part, most of them after reading the study information sheet. Reasons given for not taking part were many and varied (see Figure 1) and often non-specific, but included a number more likely to be factors in older people, for example feeling unwell at the time of contact with research officer, feeling that the study would involve too much effort for an already fatigued and unwell person, and not wanting to add to the number of existing medications taken. A factor particular to this study was a lack of recognition by potential participants that they might be under-nourished and therefore at risk of adverse outcomes, or potentially benefit from participation in a study directed at this problem. This highlights the general lack of recognition of the problem of under-nutrition in older people, both among older people themselves, and their professional and other carers [23]. We probably would have had a higher recruitment rate if studying a condition with a higher rate of recognition and concern among potential participants, for example cancer, pain, or obesity. Regarding the latter, and again highlighting the lack of recognition of under-nutrition as a problem in older people, we were surprised by how many older people contacted us after a media story or advertisement about the study asking how we might help them to *lose* weight, despite it being made clear in our information that we were looking to *increase* the nutritional state and weight of underweight, under-nourished people. Again, perhaps consistent with the low level of recognition of this problem, we recruited virtually no participants by approaching them (eg after case note review or referral from health care worker), and had much greater success when we were approached by potential participants who had become aware of the study through the media and recognised that they may have a problem.

In retrospect the information sheet (12 pages in length, 4544 words) was probably too complicated and long, and put a number of potential participants off. We might have had greater success with a a two-step process, with a shorter introductory information sheet one followed, for those expressing continued interest, by an in-person explanation and provision of the detailed information sheet mandated by our ethics committees. Attention to the layout of the information sheet is also important. Crawford Shearer et al. [24], reported documents printed in small font are difficult for visually impaired older adults. They also suggested that forms should be written in large font, divided in two sections and difficult terms should be expressed simply. Difficulty with the consent process, which involves long and complicated documents, is a common cited barrier in other studies [25,26], although we are not aware that that was the case in this study.

Reassuringly, the refusal rate in our study was similar to that reported in other studies [27,28]. In our study, many participants either did not want the pharmacological treatment or wanted to receive the nutritional supplement only. Some frail and older participants already being on multiple medications were concerned about the side effects of the proposed medications [29]. The inclusion of a pharmaceutical intervention in a randomized control trial can be a barrier to recruitment because the participants must be willing to be assigned randomly and accept treatments to which they may have been blinded [30]. Also, they must be willing to risk being assigned to placebo where some or all of the intervention, such as the nutrition supplement in this study, is available outside the study.

Despite designing the study to include home visits to make it more convenient for participants to participate, a number of participants declined because they felt that they were concerned about the length of the study and the number of visits required [31]. Older adults may lack understanding of the study protocols, have lower levels of education and be unwilling to make a time commitment [32]. Furthermore, some older persons find research studies intrusive through excessive interviews or collection of biological samples [8].

There are other strategies reported in the literature that could improve the recruitment of older people to clinical trials. Wilding et al. [33] suggested in their study that eligibility criteria should be more inclusive of those with lower cognitive functioning, mobility restrictions, and co-morbidities. They also suggested direct-mail recruitment methods and utilizing intermediaries to recruit institutionalized elderly. Kolanowski et al. [34], reported strategies that address participant and family barriers including early site evaluation and strong communication approaches with staff, participants, and families. It has also been reported that a face-to-face approach is more effective than recruiting from other sources and helped to reduce the uncertainty that older adults felt about participating in research study [2].

In the process of recruiting to this study, a number of ethical challenges were addressed including informed consent, confidentiality, and voluntary participation. Media exposure was effective in recruiting participants into the study and in creating interest in the topic and therefore, is an appropriate strategy provided that it is implemented early in the study. The researchers could have employed some other strategies to improve recruitment to this clinical trial such as direct approach to older people through community groups and activities but this was difficult in terms or our resources and time availability. It is important in designing clinical studies of older people to allow for the difficulties of recruitment, the time commitment involved and to allocate sufficient resources to the multiple strategies that might be necessary to recruit sufficient subjects.

In summary, we were able to recruit sufficient participants to complete our study of under-nourished, older, community-dwelling older people, but not as many as planned, and the recruitment period extended over 4 years, despite our use of multiple recruitment methods. There were recruitment issues related to our particular study design, but others likely to apply to most studies of older people, particularly when frail or in poor health. Media appearances and advertisements were the most effective strategy with no success from direct approaches to potential participants we had identified. It is probably important that potential subjects identify themselves as having the condition under study and that they perceive it is a problem to them – education is likely to help in this regard [35]. Multiple recruitment strategies are likely to be necessary to recruit adequate numbers in many studies involving older people. Simplification of the recruitment process as much as possible (eg not overwhelming subjects with long information sheets at first contact), establishing face-to-face contact with potential older subjects as early as possible in the recruitment process, and simplification of the study protocol to minimise burdens of the study on participants (for example reducing study visit numbers and requirements for investigations) are strategies that are likely to help with recruitment.

## Appendix 1

### ARE YOU UNDERWEIGHT?

Under-nutrition and weight loss in older people are common, but frequently overlooked. They can lead to medical problems, hospitalisation and even death.

The University of Adelaide has received funds from the Australian National Health and Medical Research Council to investigate ways of improving nutrition in older people.

- Who are we looking for?
- men and women.
- aged 65 years or older.
- at nutritional risk, ie weight loss or low body weight.
- living at home or independent living unit.

### What is involved?

A study to see if a nutritional treatment including supplement drink has benefits including reduced admissions to hospital.

If you are interested in knowing more about the study please contact us on xxxx xxxx.

This research has been approved by the Human Research Ethics Committee.
